# Obstructive Sleep Apnea Syndrome: From Symptoms to Treatment

**DOI:** 10.3390/ijerph19042459

**Published:** 2022-02-21

**Authors:** Giannicola Iannella, Giuseppe Magliulo, Antonio Greco, Marco de Vincentiis, Massimo Ralli, Antonino Maniaci, Annalisa Pace, Claudio Vicini

**Affiliations:** 1 Department of ‘Organi di Senso’, University “Sapienza”, Viale dell’Università, 33, 00185 Rome, Italy; giuseppe.magliulo@uniroma1.it (G.M.); antonio.greco@uniroma1.it (A.G.); marco.devincentiis@uniroma1.it (M.d.V.); massimo.ralli@uniroma1.it (M.R.); annalisapace90@gmail.com (A.P.); 2 Department of Medical and Surgical Sciences and Advanced Technologies “GF Ingrassia”, ENT Section, University of Catania, Via S. Sofia, 78, 95125 Catania, Italy; tnmaniaci29@gmail.com; 3 Department of Head-Neck Surgery, Otolaryngology, Head-Neck and Oral Surgery Unit, Morgagni Pierantoni Hospital, Via Carlo Forlanini, 34, 47121 Forlì, Italy; claudio@claudiovicini.com; 4 Department ENT & Audiology, University of Ferrara, Via Savonarola, 9, 44121 Ferrara, Italy

**Keywords:** obstructive sleep apnea, sleep, sleep apnea treatment, sleep apnea surgery

## Abstract

Obstructive sleep apnea (OSA) syndrome is a respiratory sleep disorder characterized by partial or complete recurrent episodes of upper airway collapse that occur during the night. The OSA manifests with a reduction (hypopnea) or complete cessation (apnea) of airflow in the upper airways, associated with breathing effort. OSA is a frequent and often underestimated pathology affecting between 2 and 5% of the middle-aged population. Typical nocturnal symptoms are the persistent snoring and awakenings with dyspnea sensation. On the other hand, diurnal symptoms could be sleepiness, headaches, asthenia, neurological disorders, and impaired personal relationships. Surgery of the velo-pharyngeal region had a huge evolution going from ablative techniques (UP3 and LAUP) to remodeling techniques of the pharyngeal lateral walls.

Obstructive sleep apnea (OSA) syndrome is a respiratory sleep disorder characterized by partial or complete recurrent episodes of upper airway collapse that occur during the night. The OSA manifests with a reduction (hypopnea) or complete cessation (apnea) of airflow in the upper airways, associated with breathing effort. OSA is a frequent and often underestimated pathology affecting between 2 and 5% of the middle-aged population [1,2,3,4]. Typical nocturnal symptoms are persistent snoring and awakenings with a dyspnea sensation. On the other hand, diurnal symptoms could be sleepiness, headaches, asthenia, neurological disorders, and impaired personal relationships [5,6,7,8,9].

During the hypopnea/apnea events, poor alveolar ventilation reduces the oxygen saturation in the arterial blood (SaO2) with a gradual increase in carbon dioxide (PaCO2) [1,5,10,11]. The direct consequence of the intermittent hypoxia could be an oxidative imbalance, with reactive oxygen species production and an inflammatory cascade activation with pro-inflammatory cytokines growth (IL2, IL4, TNF, PCR). Furthermore, an endothelial dysfunction, as indicated by increased serum levels of ET-1 and LOX-112-14, could occur. As a result of the nocturnal hypoxia and the systemic inflammations, the risks of the cardiovascular and cerebrovascular morbidities increase [1,2,3,4,5,6,7,8,9,10].

The phenotypes of OSA patients are variable depending upon the different anatomy, the collapsibility of the upper airway, the neuromuscular tone and the function sleep–wake, as well as the ventilatory control instability and the arousal threshold. Bosi et al. [5] developed a qualitative pathophysiological classification (PALM grades) by means of clinical PSG, grade of OSA severity, and therapeutic level of continuous positive airway pressure (CPAP). All of these data are a solid base for the pre-operative surgical assessment, the therapeutic recommendations, and their potential outcomes and side effects [10,11,12,13,14,15]. Drug-induced sleep endoscopy (DISE) represents another method for evaluating sites and patterns of collapse in OSA patient candidates to surgical treatment. It consists in an upper airway evaluation during a pharmacologically simulated sleep. Yu Lin et al. [15] and other literature evidence [10,11,12,13,14,15,16,17] has stated that DISE is superior to the wake-up endoscopy in identifying obstructions sites and types of collapses in the hypopharyngeal and base of the tongue regions. Recently, the use of a middle latency auditory evoked potentials (MLAEP) has been proposed as a good methodology to evaluate the correct level of sedation for patients during DISE procedures [16,17].

There are many surgical procedures proposed for the treatment of OSA. Surgery of the velo-pharyngeal region had a huge evolution going from ablative techniques (UP3 and LAUP) to remodeling techniques of the pharyngeal lateral walls [18,19,20,21]. In this scenario, barbed reposition pharyngoplasty (BRP), devised by Vicini et al. [22], showed excellent outcomes at short- and long-term follow-up [23,24]. Another surgical option, which gave optimal anatomical and functional results [25,26], is the transoral robotic surgery utilized for the base of tongue resection.

The recent introduction of hypoglossal nerve stimulation is another novel therapy for treating OSA. In a literature review, Mashaqi et al. [27] reported that it is a very effective therapy for moderate and severe OSA in patients who are intolerant to CPAP therapy.

Finally, the myofunctional therapy (MFT) with its active training of the oropharyngeal muscles has been introduced as an OSA treatment modality. The current studies demonstrate a positive effect in reducing sleep apnea, as shown by the post-therapy improvement of polysomnography values and clinical symptoms [18,19,20,21,22,23,24,25,26,27,28,29,30].
